# Atomic-Scale Optical Microscopy with Continuous-Wave Mid-Infrared Radiation

**DOI:** 10.1021/acs.nanolett.5c05319

**Published:** 2026-01-22

**Authors:** Felix Schiegl, Valentin Bergbauer, Svenja Nerreter, Valentin Giessibl, Fabian Sandner, Franz J. Giessibl, Yaroslav. A. Gerasimenko, Thomas Siday, Markus A. Huber, Rupert Huber

**Affiliations:** † Department of Physics and Regensburg Center for Ultrafast Nanoscopy (RUN), 9147 University of Regensburg, 93040 Regensburg, Germany; ‡ School of Physics and Astronomy, 1724 University of Birmingham, Birmingham B15 2TT, U.K.

**Keywords:** near-field microscopy, optical microscopy, nanoscopy, mid-infrared, near-field optical tunneling emission (NOTE)

## Abstract

Understanding matter at the most fundamental level requires optical microscopy with ever-higher spatial resolution. Scanning near-field optical microscopy (SNOM) has enabled important advances, circumventing the diffraction limit of light by confining it to the apex of a sharp metallic tip. However, the mesoscopic tip geometry restricts the spatial resolution to the nanometer scale. Here, using a conventional tabletop continuous-wave mid-infrared laser and intensity-based detection we observe optical signals modulated on Ångstrom length scales, consistent with light emission from atomically confined tunneling currents. The emergence of near-field optical tunneling emission (NOTE) — considered a strong-field excitation process — under continuous-wave driving is remarkable, as it typically requires ultrashort high-intensity laser pulses. Further, we find that anharmonic tip oscillation can influence the signal and propose strategies to mitigate this effect. Our findings enable the use of this tunneling-mediated contrast mechanism with standard optical setups, establishing a pathway to optical imaging with unprecedented resolution.

## Graphical abstract

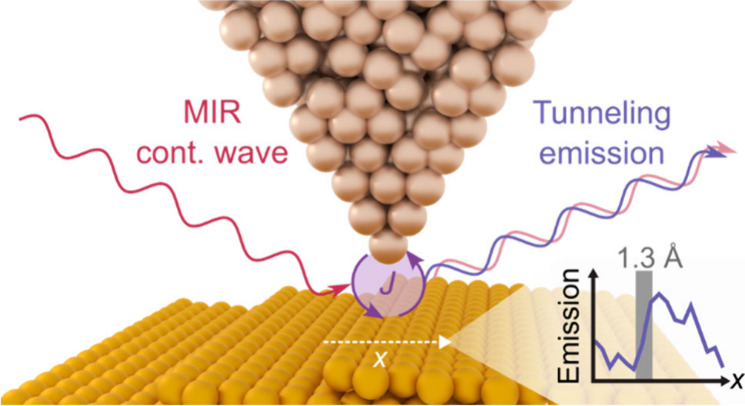

To deepen our understanding of matter at its most fundamental level, advancing optical microscopy to the shortest possible length scales is crucial. While super-resolution techniques have successfully overcome the diffraction limit of light,^1^ they typically rely on fluorescent labeling and are therefore often incompatible with solid-state systems. An alternative pathway has been the use of plasmonic nanostructures, where extreme confinement of optical fields in picocavities has enabled single-molecule spectroscopy.^2^ However, this approach remains restricted, as it cannot be applied effectively at spectral ranges far from plasmonic resonances and is usually unsuitable for imaging applications. Near-field optical microscopy (SNOM) has proven to be a universal method of optical imaging beyond the diffraction limit, where sharp metallic tips act as antennas that confine light in an ultrabroad frequency range.^3,4^ Despite its versatility^5–14^ the spatial resolution of SNOM has so far been limited to roughly ten nanometers.^4^ Recent progress, enabled by nanometer-scale tip oscillation amplitudes using self-sensing quartz sensors (e.g. qPlus^15^) in ultrahigh vacuum, has demonstrated a resolution improvement down to the single-nanometer scale in the visible spectral range using plasmonic tips.^16^

Achieving true atomic resolution, however, requires a fundamentally different contrast mechanism, in which the carrier wave of light drives atomically confined tunneling currents between the tip and the sample. By electrically detecting the rectified component of these currents, lightwave-driven scanning tunneling microscopy has enabled insights into ultrafast atomic-scale dynamics.^17–20^ Recently, we have developed a more direct way to read out these a.c. tunneling currents by detecting the light emitted from them, thereby enabling all-optical microscopy with atomic-scale spatial resolution (Figure 1a).^21^ Reaching the strong-field regime where the carrier wave of light can coherently drive electrons **—** enabling lightwave electronics^22^ **—** has so far only been possible with ultrafast tabletop lasers or free-electron lasers.^23^ This requirement has posed a major barrier to broader adoption of this contrast mechanism within the SNOM community, where continuous-wave illumination (Figure 1b) and intensity-based detection are an established standard.^4^

**Figure 1:**
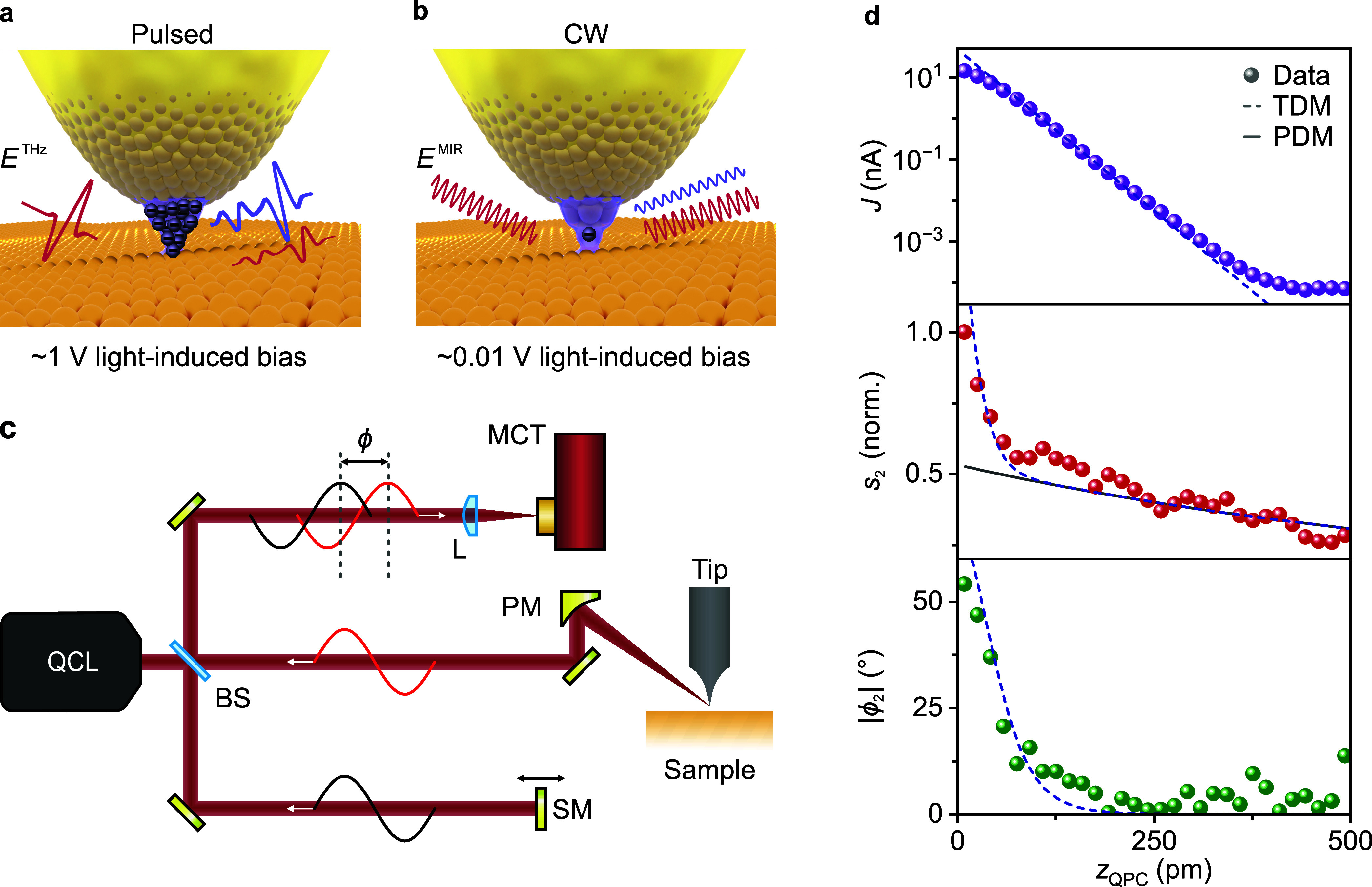
Near-field optical tunneling emission (NOTE) with mid-infrared (MIR) continuous-wave (cw) radiation. (a) Schematic of ultrafast terahertz (THz) NOTE microscopy. THz pulses are focused onto a sharp metallic tip placed within atomic distance to an Au(111) surface. The transient electric field induced lightwave bias on the order of 1 V drives electrons back and forth the tunneling junction, leading to a NOTE waveform (violet) being emitted alongside the conventional near-field signal (red). (b) Schematic of NOTE microscopy with cw MIR radiation, where the peak lightwave-induced bias is several orders of magnitude smaller, leading to much lower lightwave-induced tunneling currents. (c) Experimental setup: Radiation from a commercial quantum cascade laser (QCL) is focused onto a sharp metallic tip placed above an Au(111) sample using a parabolic mirror (PM). The backscattered light is collimated and, via a beamsplitter (BS), superimposed with a split-off portion of the beam that is harmonically modulated in its phase ϕ using a piezo-driven shaker mirror (SM). Using this pseudoheterodyne detection scheme, the amplitude and phase of the scattered light detected with a mercury-cadmium-telluride photodiode (MCT) can be extracted (see Supporting Information Section 2). (d) Signals originating from the tip-sample junction as a function of the tip-sample distance *z*_QPC_ at the point of closest approach (oscillation amplitude *A* = 200 pm), calibrated via the expected value for the tunneling current *J* at the quantum point contact (QPC). Top panel: tunneling current *J* (d.c. bias voltage *V*_dc_ = 3 mV). Central and bottom panel: simultaneously acquired near-field amplitude *s*_2_ (red) and magnitude of the relative phase |ϕ_2_| (green), demodulated at the second harmonic of the tip oscillation frequency. The dashed lines correspond to fits using the tunneling dipole model (TDM, see Figure 2), while the solid line shows the contribution of only the point dipole model (PDM).

Here, we demonstrate that, surprisingly, tunneling-current-mediated contrast can be accessed even with a conventional, continuous-wave mid-infrared (MIR) setup based on a tabletop quantum cascade laser and intensity-based detection, making atomic-scale optical microscopy broadly compatible with existing near-field platforms. We observe optical signals that emerge only at atomic-scale distances, in line with the near-field optical tunneling emission (NOTE) mechanism rather than conventional SNOM. We further identify potential artifacts that can arise under these unique experimental conditions and implement strategies to exclude their influence on the observed features. Our findings open the door to atomic-scale imaging using tools that are already established in the near-field community, offering a practical route to optical access on the level of individual atoms.

We use a well-established optical setup for near-field microscopy based on a commercial quantum cascade laser combined with pseudoheterodyne detection^24^ and couple it to a commercial scanning probe microscope (see Figure 1c and Supporting Information, Sections 1 and 2). By mounting our tips to stiff qPlus sensors (ref 15, Supporting Information Section 3), we achieve precise control over the tip motion, allowing for reduction of the oscillation amplitude by several orders of magnitude: from the ∼10–100 nm range typical of conventional cantilever-based SNOM, to 100 pm. Additionally, frequency-modulation noncontact atomic force microscopy (AFM) enables us to stabilize the tip-sample separation with atomic-scale precision. Moreover, our experimental configuration provides simultaneous access to the scattered optical fields and the rectified tunneling currents between tip and sample, allowing for distinction of effects based on tunneling and conventional near-field scattering.

First, the dependence of the scattered optical signal on the tip-sample distance *z* is explored. To this end, we approach an Au(111) surface (oscillation amplitude *A* = 200 pm, d.c. bias voltage *V*_dc_ = 3 mV) while simultaneously recording the tunneling current *J*, the near-field amplitude *s*_2_, and the magnitude of the relative optical phase of the scattered light |ϕ_2_| (see Figure 1d). To calibrate the absolute tip-sample distance *z*_QPC_ at the point of closest approach during the oscillation cycle of the tip, the exponentially decaying tunneling current is extrapolated to the value expected in quantum point contact (QPC, Supporting Information Section 4).^25^ The measured near-field amplitude *s*_2_ and the magnitude of the phase |ϕ_2_| are shown in the central and bottom panel of Figure 1d. At tip-sample distances larger than *z*_QPC_ = 150 pm, the observed behavior aligns well with what is expected for a conventional SNOM signal: *s*_2_ increases gradually with decreasing *z*_QPC_, and |ϕ_2_| remains constant within the noise floor of the detection. Intriguingly, at distances below 100 pm, *s*_2_ rises much more steeply, accompanied by a distinct phase shift of ∼50°. This distance dependence of the optical signals cannot be reproduced by the established point dipole model (PDM^26^), which predicts no pronounced phase shift and a smaller increase in amplitude at small tip-sample separations (Figure 1d, solid line). Instead, the observed features in *s*_2_ and |ϕ_2_| are consistent with light emission from a.c. tunneling currents which are driven by the electric field of light and can be described by a straightforward tunneling dipole model (TDM, Figure 1d, dashed lines; Supporting Information Section 5).

In this model, the incident light not only induces a near-field dipole *p* ^NF^ at the apex of the tip, as in conventional SNOM models, but also acts as a transient bias *V*_lw_(*t*), that adds to the applied d.c. bias *V*_dc_. For strong fields and small tip-sample separations, this drives a tunneling current based on the *I*-*V*-curve of the tip-sample system as measured with scanning tunneling spectroscopy (see Figure 2a, purple), evaluated at the time-dependent bias values *V*(*t*) = *V*_dc_ + *V*_lw_(*t*) (Figure 2a, yellow). As a result, the tunneling current becomes time dependent (Figure 2b, purple) and induces an additional tunneling dipole *p* ^*J*^ through charge accumulation (Figure 2b, red). Assuming instantaneous tunneling, the lightwave-induced bias, which is proportional to *p* ^NF^ (Figure 2b, blue), and the tunneling current are in phase. However, due to the time-integrating process of charge accumulation via tunneling, the induced tunneling dipole *p* ^*J*^ shows a π/2 phase shift (Figure 2b).

**Figure 2:**
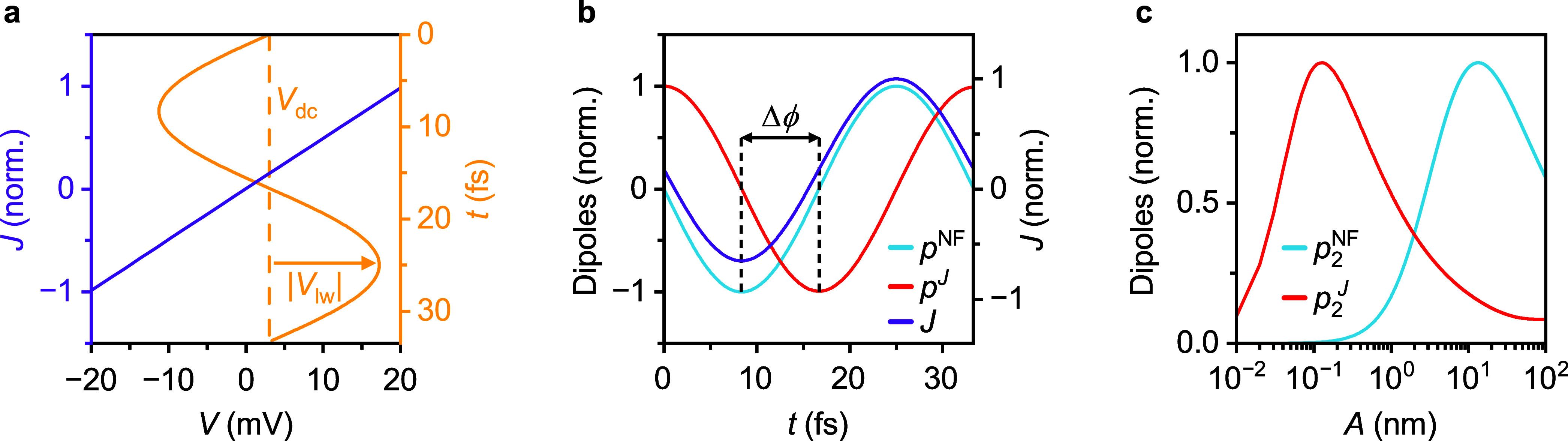
Tunneling dipole model (TDM) of the MIR cw NOTE signal. (a) Modeling the time-dependent tunneling currents. The mid-infrared radiation acts as a time-dependent bias voltage *V*_lw_(*t*) that adds to the applied d.c. voltage (*V*_dc_ = 3 mV, dashed yellow line). The total bias *V*(*t*) = *V*_dc_ + *V*_lw_(*t*) (yellow line) drives a time-dependent tunneling current *J* according to the conductance of the gold substrate (violet). (b) Temporal evolution of the near-field dipole *p* ^NF^ and tunneling dipole *p* ^*J*^ during one oscillation cycle of the driving field simulated with the TDM. *p* ^*J*^ (red) is formed as a time integral over the tunneling current *J* (violet), resulting in a phase shift Δϕ = π/2 (see vertical black dashed lines) compared to *p* ^NF^ (blue, calculated via the point dipole model (PDM)). (c) Calculated oscillation amplitude dependence of the dipoles *p*_2_^NF^ and *p*_2_^*J*^, demodulated at the second harmonic of the oscillation frequency. Picometric oscillation amplitudes (*A* < 1 nm) favor the contribution of *p*_2_^*J*^ over *p*_2_^NF^. By extracting the sum of *p*_2_^NF^ and *p*_2_^*J*^ for a constant value of the oscillation amplitude *A* = 200 pm while varying the average tip-sample separation, we reproduce the experimentally observed decay of the optical signals as well as the emerging phase shift (dashed lines in Figure 1d). Simultaneously, the modeled tunneling current *J* also matches the experimental data.

Both time-dependent dipoles radiate into the far field, directly imprinting their relative phase shift onto the measured signals. By incorporating the exponential distance-dependence of the tunneling current, the model also captures the influence of varying tip-sample separation, thereby allowing us to incorporate the effect of the tip oscillation. Thereby, the influence of the amplitude set point *A* on the relative magnitude of the experimentally accessible dipoles, *p*_2_^NF^ and *p*_2_^*J*^, obtained by demodulating at the second harmonic of the oscillation frequency, can be calculated (Figure 2c). While for conventional oscillation amplitudes (∼10–100 nm), the relative contribution of the tunneling-induced signal is negligibly small, operating at subnanometer amplitude set points drastically enhances the relative charge-transfer-induced signal (compare Figure 2c).

Comparing this model with the experimental data in Figure 1d, it is obvious that the TDM (dashed lines) does not only reproduce the gradual increase of the scattered optical near-field signal with decreasing distance but simultaneously also the rapid rise in signal at tip-sample separations *z*_QPC_ ≲ 150 pm. Simultaneously, the model accurately captures the corresponding phase shift as well as the tunneling current as a sum of the d.c. current *J*_dc_ and the lightwave-driven current *J*_lw_, confirming that the observed atomic-scale signal originates from tunneling emission. The emergence of light emission from coherent electron driving resembles the Brunel effect in atoms,^27^ but here the subcycle current traverses the tip-sample junction, confining the effect to a single atomic-scale gap. This observation comes as a surprise, as strong-field effects are not usually accessible with a tabletop continuous-wave source. In Keldysh’s model of tunnel ionization^28^ **—** conceptualized for gases but commonly also applied to solids **—** an electron tunnels through an approximately triangular potential barrier whose width depends on the applied electric field, making tunneling processes only relevant for strong fields. In the present experiment, however, the physical situation is fundamentally different. The tunneling occurs across the vacuum gap between tip and sample,^29^ whose width is primarily determined by the geometric tip-sample separation. The role of the optical field is therefore not to reshape the barrier itself but rather to induce a transient bias across an already narrow tunneling junction. This transient lightwave-induced bias modulates the energetic alignment of the tip and sample Fermi levels, thereby enabling tunneling even at moderate field strengths. Owing to the local field enhancement at the tip apex, we estimate the lightwave-induced bias to reach a few millivolts (Supporting Information Section 6). At the closest tip-sample distance of *z*_QPC_ = 9 pm, our model predicts that on average one electron tunnels across the junction in 100 optical cycles. In the experiment, the optical excitation power (5 mW) lies within the upper range reported to be compatible with a stable tip-sample junction in low-temperature tip-based measurements,^30^ and we likewise find that further increases in power lead to reduced stability, thereby constraining the achievable lightwave-induced bias. As a consequence, extremely small tip-sample distances (*z*_QPC_ ≲ 50 pm in this experiment) are necessary to achieve sufficiently strong lightwave-driven tunneling currents for the near-field optical tunneling emission to dominate the scattering response. Yet, it shows that atomic-scale tunneling can be accessed with standard continuous-wave optical setups, paving the way toward more widespread use of tunneling-current-mediated optical contrast mechanisms.

Having used the phase shift in the optical response to confirm tunneling emission as the dominant contrast mechanism at small tip-sample distances and oscillation amplitudes, the signal quality can be improved by switching from pseudoheterodyne to intensity-based detection, sometimes also referred to as self-homodyne detection.^16^ At the small amplitude set points used in this study (*A* < 1 nm), we find that the measured intensities *I*_2_ demodulated at the second harmonic of the oscillation frequency are virtually background-free (Supporting Information Section 7). Moreover, the improved stability of the signal due to the simpler setup results in enhanced long-term stability. This allows us to decrease the oscillation amplitude by a factor of 2 to only 100 pm, further enhancing the tunneling emission contribution.

As this signal stems from atomically confined tunneling currents, it should also feature atomic-scale lateral resolution. To test this, we perform a 3 nm by 3 nm scan on a vicinal gold surface^31^ in AFM feedback (oscillation amplitude *A* = 100 pm, frequency shift set point Δν_set_ = −1.7 Hz). The topography (Figure 3a) clearly shows the characteristic terrace-like structure consisting of periodically spaced monatomic steps, which are known to affect the surface electronic structure of the material.^32^ The simultaneously recorded scattered intensity *I*_2_ exhibits a modulation on the same lateral scale (see Figure 3b). In particular, *I*_2_ displays enhanced intensity at the atomic step edges. To quantitatively assess the spatial confinement of the optical signal, we extract a line profile from the two-dimensional *I*_2_ map and analyze a representative edge feature (line profile in Figure 3b, inset). By fitting the edge with an error function, we determine the lateral distance between the 90% and 10% intensity level of the step edge, revealing a modulation on the length scale of 128 pm. As a consistency check, we analyze eight additional step edges, yielding a mean modulation width of 130 pm and a standard deviation of 35 pm (Supporting Information Section 8). Remarkably, the spatial distribution of the simultaneously measured tunneling current *J* for minimized d.c. bias (Figure 3c) closely mirrors that of *I*_2_, exhibiting the same stripe-like modulation and enhanced signal at the descending edges of atomic steps. This strong similarity between *I*_2_ and *J* provides further compelling evidence that the scattered light originates from tunneling electrons.

**Figure 3:**
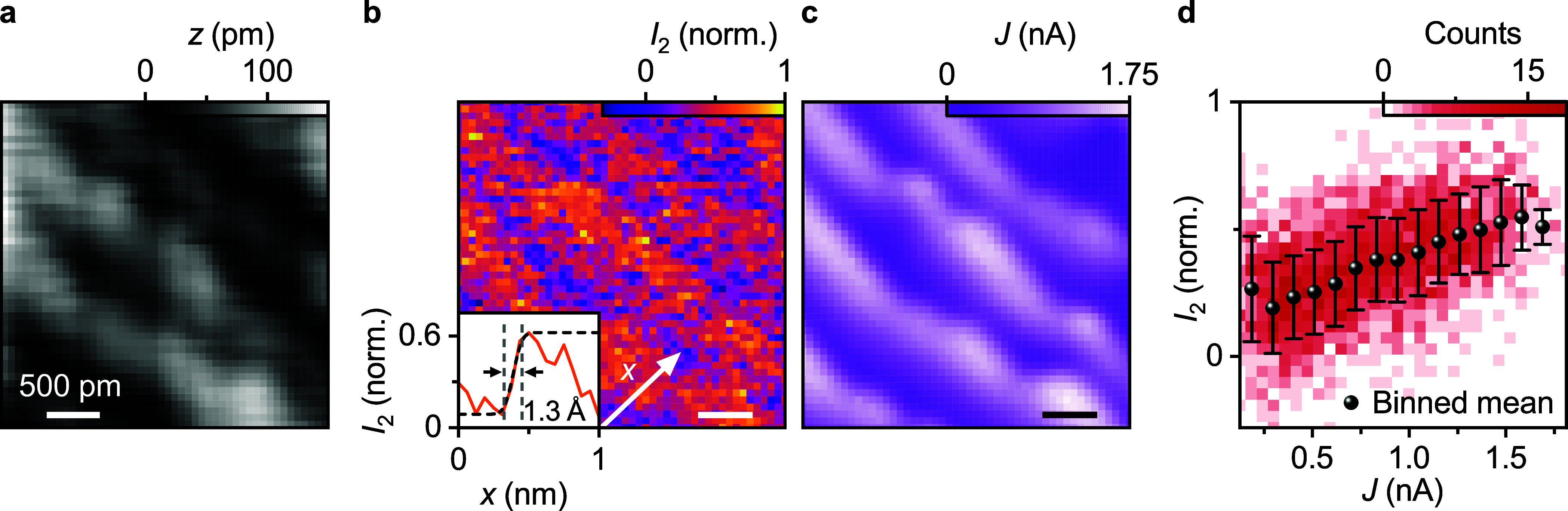
Optically resolving atomic-scale features using MIR cw radiation. (a) Topography of a vicinal gold surface imaged with atomic force microscopy (AFM) (oscillation amplitude *A* = 100 pm, frequency shift set point Δν_set_ = −1.7 Hz), revealing characteristic monatomic steps. (b) Simultaneously acquired map of the scattered intensity *I*_2_, demodulated at the second harmonic of the tip oscillation frequency. A clear contrast emerges with enhanced intensity at the atomic step edges. The inset shows a linecut of *I*_2_ along *x*. Fitting a step in the signal with an error function (black dashed line), we find a spatial modulation on the length scale of 1.3 Å using a 90%–10% criterion. (c) Simultaneously acquired map of the tunneling current *J*, recorded under minimized d.c. bias, where a close resemblance to the *I*_2_ map is found. (d) Two-dimensional histogram representing the pixelwise correlation between the current *J* and scattered intensity *I*_2_, indicating a linear correlation between the two quantities. The color map indicates the number of pixels whose value pairs fall into each bin. The black spheres depict the data points binned exclusively along the *J*-axis, while the error bars represent one standard deviation. For clarity, the AFM topography was line-leveled and smoothed using a Gaussian filter.

A pixel-by-pixel correlation analysis between *I*_2_ and *J* (Figure 3d) reveals a clear correlation, with higher values of scattered intensity consistently associated with regions of increased tunneling current. Binning the data along the *J*-axis and computing the mean of *I*_2_ for each bin (Figure 3d, black spheres) reveals an approximately linear dependence of *I*_2_ on *J*. This correlation further confirms that both signals originate from the same picometric contrast mechanism, likely a modulation of the local tunneling probability, even though a partial influence of the sample’s modulated topography due to small variations in tip-sample distance during scanning cannot be completely ruled out within the sensitivity of our measurement. Generally, however, we find a clear correlation between the optical signal and the tunneling currents that are conventionally used to resolve molecules and single adatoms, suggesting that our all-optical method could perform equally well.

Having reduced the oscillation amplitude to 100 pm and changed the detection scheme to enhance the signal, the signal could potentially be further increased by optimizing the tip-sample distance for the reduced oscillation amplitude. Therefore, we perform another two-dimensional scan with a frequency shift set point of −1.8 Hz, effectively approaching the tip further to the sample. The AFM topography (Figure 4a) and the scattered intensity *I*_2_ (Figure 4b) show an overall similar behavior to the previous scan. However, apart from the previously observed variation along the step edges, *I*_2_ displays additional hotspots of elevated intensity. To find the origin of this locally confined signal enhancement, we trace the oscillation of our scanning probe sensor by measuring the voltage signal generated by the piezoelectric quartz sensor, on which the tip is mounted. Directly accessing this signal at the second harmonic of the tip oscillation frequency *A*_2_ via lock-in detection (Figure 4c, see Supporting Information Section 9 for the exact calibration procedure), we find that *I*_2_ and *A*_2_ show the same hotspots (Figure 4b and 4c). This suggests that the hotspots in *I*_2_ are linked to a locally occurring anharmonic motion of the tip^33,34^ at these small tip-sample distances. The two-dimensional histogram of the pixelwise correlation of *I*_2_ and *A*_2_ (Figure 4d) confirms this interpretation, showing that for *A*_2_ values over 4 pm *I*_2_ seems to be increasing with *A*_2_ — a relation discernible only thanks to the remarkable stability and sensitivity of our probe.

**Figure 4:**
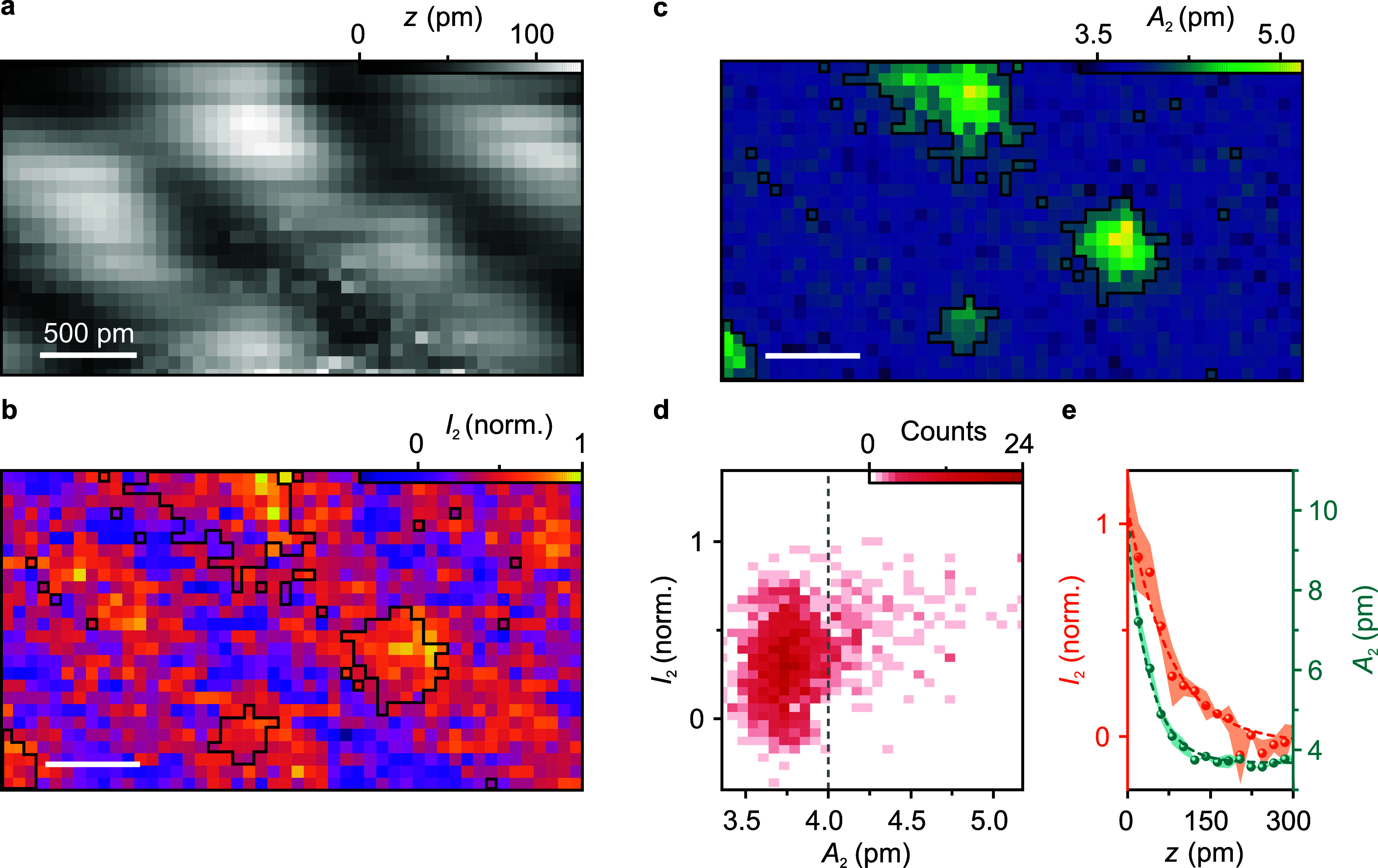
Optical imaging at the mechanical limit. (a) Topography of the vicinal gold surface, measured in AFM feedback mode (*A* = 100 pm, Δν_set_ = −1.8 Hz). (b) Simultaneously recorded scattered intensity *I*_2_, where a clear modulation can be seen. (c) Simultaneously acquired map of the mechanical tip oscillation amplitude *A*_2_, demodulated at the second harmonic of the oscillation frequency. The scan shows local peaks of *A*_2_, indicating anharmonicity of the tip oscillation. (d) Two-dimensional histogram representing the pixelwise correlation between *I*_2_ and *A*_2_, where the color map indicates the number of value pairs that fall into each bin. High values of *A*_2_ tend to coincide with high values of *I*_2_. The highlighted areas in b and c show the data points to the right of the gray dashed line, where *A*_2_ > 4 pm. (e) Comparison of the distance dependence of the intensity *I*_2_ and the second harmonic oscillation amplitude *A*_2_, revealing different decay lengths for both signals. The dashed lines show exponential fits of the data. The experimental data in panels d and e were averaged over the forward and backward direction of the scan with the shaded area representing one standard deviation.

Quantitatively, the linear correlation can be described by the Pearson correlation coefficient *r*, which ranges from −1 (perfect negative linear correlation) to +1 (perfect positive linear correlation), with *r* = 0 indicating no linear relationship.^35^ For this data set, we obtain *r* = 0.305, indicating a weak to moderate positive correlation. This is a crucial insight showing that for all measurements performed with similar setups in the future, measuring the harmonicity of the tip oscillation is imperative for reliable results at such small tip-sample distances. Since for *A*_2_ < 4 pm no obvious correlation between *A*_2_ and *I*_2_ can be observed, we define a threshold and exclude all data points with second harmonic amplitude contributions of *A*_2_ > 4 pm (marked regions in Figure 4b,c and to the right of the dashed line in Figure 4d). Reassessing the correlation between *I*_2_ and *A*_2_ using only the remaining 86% of data, we obtain a Pearson coefficient of *r* = 0.096, hinting toward no pronounced linear correlation.^35^ This indicates that, despite the localized enhancement of *A*_2_, the optical signal *I*_2_ remains unaffected across the vast majority of locations. Notably, applying the same thresholding procedure to the scan shown in Figure 3 would result in the exclusion of only 10 data points, retaining 99.6% of the scan (Supporting Information Section 10). To exclude the possibility that variations in *A*_2_ account for the observed optical behavior in Figure 1, we performed an analogous measurement while tracking *A*_2_, which yields qualitatively similar optical signals while *A*_2_ remains flat (Supporting Information Section 11).

Thus, one can reliably extract the local optical response by carefully monitoring the tip oscillation and only evaluating regions where the local amplitude *A*_2_ remains well-behaved. Alternatively, anharmonic tip motion can be effectively suppressed by choosing a suitable tip-sample distance, balancing signal strength and well-behaved tip motion. To this end, we investigate the dependence of *I*_2_ and *A*_2_ on the tip-sample distance *z* at one position in detail (Figure 4e). *I*_2_ increases exponentially with a nonzero signal already present at the highest tip-sample distance of 300 pm and a 1/e decay length of 77 pm. The simultaneously recorded data for *A*_2_ show a similar behavior, however, first remaining below the noise threshold (∼4 pm) for larger separations and only exponentially increasing at distances below ∼120 pm with a 1/e value of 40 pm. Hence there is a finite tuning range of tip-sample distances where *I*_2_ is already nonzero while *A*_2_ remains negligible, thus establishing a practical regime where tunneling-current-mediated optical contrast can be accessed even with standard tabletop continuous-wave laser sources.

In conclusion, we combined an ultrahigh-vacuum scanning probe microscope capable of picometric tip control with a standard optical setup for near-field microscopy using a commercial quantum cascade laser and pseudoheterodyne photodiode detection. At atomic-scale tip-sample distances, we observed a signal exhibiting the two hallmark features of NOTE: a picometric vertical decay and a distinct phase shift relative to the conventional near-field response **—** both consistent with our TDM. We achieved atomic-scale optical microscopy on a vicinal gold surface, where the scattered intensity showed a clear positive correlation with the tunneling current, in line with the NOTE mechanism. At extremely small tip-sample distances, a second harmonic contribution in the tip’s oscillation emerges **—** an effect that can potentially give rise to artifactual signals. By closely monitoring the tip’s mechanical behavior, we identified a stable operational regime in which the tunneling-related contrast mechanism can be reliably used to extract signal modulations on the Ångstrom length scale with artifacts from anharmonic tip oscillation effectively ruled out.

Our findings represent an important step toward the broader adoption of atomic-scale optical microscopy, combining the spatial resolution of scanning probe techniques such as STM and AFM with the frequency selectivity of near-field microscopy. This opens the door to extend the toolbox of atomic-scale spectroscopy: By tuning the optical frequency, one could map the local dielectric function, while sweeping the tip-sample bias may allow the optical signal to probe differential conductance in this highly nonequilibrium state. As NOTE does not rely on a net current to flow, it is compatible even with insulating samples, where this concept could be particularly intriguing: The sensitivity to all tunneling carriers **—** not only those that are rectified **—** could enable the investigation of tunneling processes that remain hidden to conventional electronic detection. More broadly, our results contribute to a deeper understanding of atomic-scale interactions in tip-based optical measurements **—** a key to advancing nanoscale spectroscopy and imaging.

## Supplementary Material



## Data Availability

The data sets generated during and/or analyzed during the current study are available from the corresponding author upon request.

## Notes

(1) Low-temperature near-field microscopy setup; (2) Optical setup for mid-infrared NOTE microscopy; (3) Details on the tips used in the experiment; (4) Calibration of the absolute tip-sample distance; (5) Details of the tunneling dipole model; (6) Estimation of the lightwave-induced bias under cw illumination; (7) Background-free near-field detection at small oscillation amplitudes; (8) Statistical analysis of the modulation length of the NOTE signal; (9) Calibration of the tip oscillation amplitude; (10) Excluding anharmonicity artifacts in the 2D scan of Figure 3; (11) Excluding anharmonicity artifacts as the origin of the optical signals in Figure 1 (PDF)
