# Supplementary material for: Atomic-Scale Optical Microscopy with Continuous-Wave Mid-Infrared Radiation
Source: Nano Lett. 2026 Jan 22;26(5):1689–96. doi: 10.1021/acs.nanolett.5c05319 (PMC12904093; doi:10.1021/acs.nanolett.5c05319)
Supplement: Supplementary file 1 [file nl5c05319_si_001.pdf]

Supporting information

Atomic-scale optical microscopy with continuous-wave mid-infrared radiation

Felix Schiegl[1,‡], Valentin Bergbauer[1,‡], Svenja Nerreter[1], Valentin Giessibl[1], Fabian Sandner[1], Franz J. Giessibl[1], Yaroslav A. Gerasimenko[1], Thomas Siday[2,], Markus A. Huber[1,*], and Rupert Huber[1]*

[1]Department of Physics and Regensburg Center for Ultrafast Nanoscopy (RUN), University of Regensburg, 93040 Regensburg, Germany

[2]School of Physics and Astronomy, University of Birmingham, Birmingham B15 2TT, U.K.

[‡]These authors contributed equally to this work

**Correspondence should be addressed to these authors*

1. Low-temperature near-field microscopy setup

The experiments were performed using a UNISOKU USM-1400 ultrahigh-vacuum, low-temperature scanning probe microscope equipped with custom parabolic mirrors (numerical aperture ≈ 0.4) for focusing and collecting light at the tip. For qPlus AFM/STM operation, a metallic wire was mounted on the prong of the qPlus sensor [15] and subsequently sharpened by electrochemical etching. Further details on the tips used in the experiments are provided in Section 3. The current flowing between tip and sample is amplified with a transimpedance preamplifier (Femto DLPCA-200). Since the tip-oscillation frequency (~20 kHz) is much higher than the preamplifier bandwidth (-3 dB at 7 kHz in Fig. 1, -3 dB at 1.1 kHz in Fig. 3), the detected signal corresponds to the current integrated over the oscillation cycle. The experiments shown in Figure 1 were performed at a temperature of 10 K, while the data shown in Figures 3 and 4 were measured at 20 K.

2. Optical setup for mid-infrared NOTE microscopy

The quantum cascade laser (Daylight Solutions MIRcat) was operated at a wavelength of 10 μm. For all experiments, 5 mW of power were focused onto the tip. To extract both the amplitude and phase of the scattered near-field signal, we use a pseudo-heterodyne detection scheme, originally introduced by Ocelic et al. [24]. This technique is based on a Michelson-type interferometric approach, where a portion of the light is split off to form a reference arm, which is periodically modulated in its length ($f_{\mathrm{ref}} \approx 432$ Hz). Due to this modulation, the measured signal contains components at frequencies $f_{n,m} = nf_{\mathrm{tip}} + mf_{\mathrm{ref}}$, where n indexes the harmonics of the tip oscillation ($f_{\mathrm{tip}} \sim 20$ kHz) and m indexes the sidebands originating from the reference modulation. In our experiments, the first two sidebands m = 1 and m = 2 are used, allowing the simultaneous

retrieval of the scattered near-field amplitude s_2 and phase ϕ_2 demodulated at the second harmonic of the tip-oscillation frequency.

3. Details on the tips used in the experiment

For the experiments shown in Figure 1 of the main text, a platinum-iridium tip was used, while Figures 3 and 4 show results obtained with a tungsten tip, both fabricated via electrochemical etching. For the latter, tungsten oxides were removed by treatment with concentrated hydrofluoric acid before insertion into the microscope. The mesoscopic shape of both tips was characterized by scanning electron microscopy prior to insertion (Fig. S1), yielding a radius of curvature at the apex of r = 7.5 nm and r = 10 nm, respectively. However, as typical in STM, the relevant microscopic apex structure is not directly accessible and is generally unknown at the single-atom level – particularly after *in situ* conditioning and shaping during STM operation. The essential requirements to access the NOTE signal are (i) sufficient field enhancement at the apex, which is governed primarily by the macroscopic tip shape and (ii) a well-defined and mechanically stable single-atom termination, which determines the spatial confinement through wavefunction overlap. After obtaining a well-defined macroscopic geometry through electrochemical etching, we condition the apex *in situ*, following standard STM preparation procedures, to achieve a single-atom apex [29].

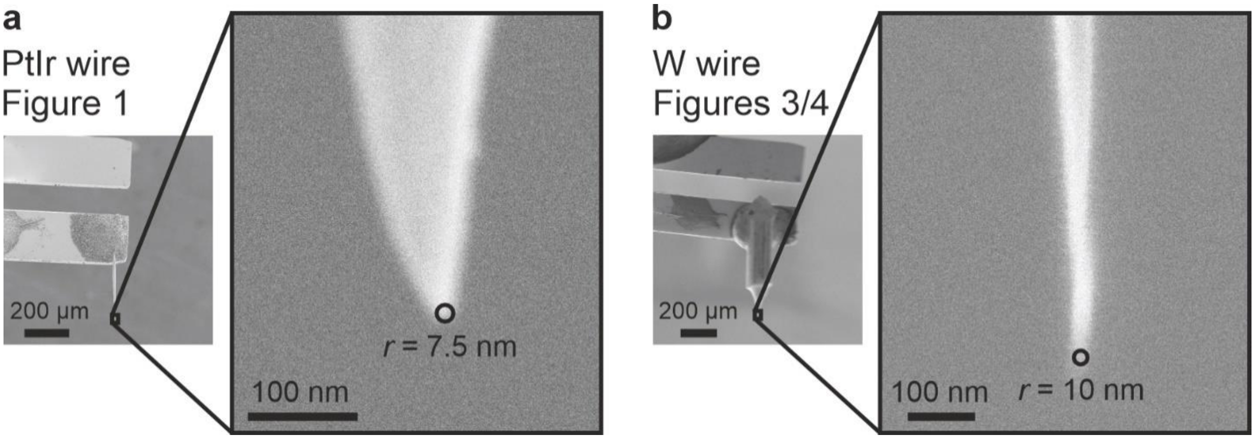

Figure S1. Scanning electron microscopy (SEM) images of the tip apices. (a) SEM image of the platinum-iridium wire and zoom-in onto the apex of the tip used for the measurements shown in Figure 1 of the manuscript. (b) SEM image of wire and apex of the tungsten tip used for the measurements presented in Figures 3 and 4 of the main text.

4. Calibration of the absolute tip-sample distance

To determine the absolute tip-sample distance with atomic precision, we utilize the experimentally acquired tunneling current and relate it to the expected value for the quantum point contact (QPC). We define $z_{\mathrm{QPC}} = 0$ as the distance at which the tip contacts the sample at the lower turning point of its oscillation cycle, enabling ballistic electron transport across the junction. This results in a current $I_0 = G_0 V$, where G_0 is the conductance quantum and V is the applied bias voltage [36]. To calibrate the tip-sample distance based on the tunneling current, we fit the experimentally recorded current data using the exponential function $I = I_0 \mathrm{e}^{-2\kappa z}$. From this fit, we then extract the decay constant κ. Since the tunneling probability is modulated during the oscillation cycle and the bandwidth of the current preamplifier (-3 dB at 7 kHz for the data shown in Fig. 1) is significantly lower than the tip's oscillation frequency (~ 20 kHz), the current measured in the experiment is averaged over the oscillation cycle and must first be related to the instantaneous current at the

point of closest approach [15]. We then use this calculation to extrapolate the experimentally measured tunneling current to the value expected for the QPC, directly yielding the corresponding absolute tip-sample distance z_{QPC} as shown in Figure 1d of the main text. We note that this evaluation is subject to uncertainties, with the dominant contributions arising from the calibration of the z-scanner piezo and the d.c. bias voltage. As conservative upper limits, we assume an uncertainty of 20% for the z-scanner piezo calibration and 30% for the d.c. bias voltage. Accounting for these two errors results in an upper limit of 18.6 pm and a lower limit of -1.9 pm for the smallest experimentally observed distance to the QPC in Fig. 1d.

5. Details of the tunneling dipole model

The scattered optical signal is modeled as arising from two distinct dipolar contributions: a near-field dipole p^{NF} and a tunnelling dipole p^{J}, formed by charges accumulated through lightwave-driven tunneling. The near-field dipole p^{NF} is described using the well-established PDM, in which the tip is approximated as a sphere of radius r_{NF} interacting with its image charge in the sample [26]. Accordingly, the near-field dipole is given by

$$p^{\mathrm{NF}}(t) = \alpha_{\mathrm{eff}} \times E_{\mathrm{light}}(t), \tag{5.1}$$

with an effective polarizability

$$\alpha_{\mathrm{eff}} = \frac{\alpha}{1 - \frac{\alpha\beta}{16\pi(r_{\mathrm{NF}} + h)^3}}. \tag{5.2}$$

Here, $E_{\mathrm{light}}(t) = \hat{E}_{\mathrm{light}} \sin(2\pi f_{\mathrm{light}} t)$ is the incident electric field, h is the tip-sample distance, $\beta = \frac{\varepsilon_{\mathrm{s}}-1}{\varepsilon_{\mathrm{s}}+1}$, and $\alpha = 4\pi r_{\mathrm{NF}}{}^3 \frac{\varepsilon_{\mathrm{t}}-1}{\varepsilon_{\mathrm{t}}+2}$, where ϵ_{t} and ϵ_{s} are the dielectric functions of tip and sample.

The lightwave-driven bias is proportional to the near-field dipole:

$$V_{\text{lw}}(t) = \hat{V}_{\text{lw}} \frac{p^{\text{NF}}(t)}{p^{\text{NF}}_{\text{max}}}. \tag{5.3}$$

We choose the magnitude of the lightwave-driven bias $\hat{V}_{\text{lw}}$ in agreement with finite-element simulations of the local field enhancement, an established approached used in lightwave-driven scanning tunneling microscopy (see Supporting Information Section 6).

Inserting $V_{\text{lw}}(t)$ into the experimentally acquired current-voltage characteristic $J(V)$ (see Fig. 2a of the main text) results in the instantaneous tunneling current $J(t)$. Integrating over time yields the time-dependent charge accumulated through tunneling,

$$Q(t) = \int_0^t J[V_{\text{lw}}(t')]\mathrm{d}t' = \int_0^t J(t')\mathrm{d}t'\,. \tag{5.4}$$

Finally, the tunneling dipole moment is obtained as

$$p^J(t) = \left(2h + 2r_J\right) \times Q(t), \tag{5.5}$$

where r_J is the effective tip radius for the tunneling dipole, making $2h + 2r_J$ the distance between the accumulated charge in the tip and its image charge in the sample.

To enable direct comparison with the experimentally measured optical signals, demodulation at harmonics of the tip oscillation frequency is implemented. For the near-field dipole p^{NF}, the distance dependence is intrinsically contained in the effective polarizability. For the tunneling dipole p^J, it is implemented by scaling the current-voltage characteristic $J(V)$ according to the distance dependence of the measured tunneling current. The resulting modulated dipoles are Fourier-analyzed over one tip oscillation cycle to extract the harmonic components. This procedure is repeated for different tip-sample separations to model the retraction curves, where p^J and p^{NF} are added to obtain the optical signal for distance. The modelled curves in Fig. 1d have been obtained with $r_{\text{NF}} = 5$ nm, $r_J = 150$ pm, and $\hat{V}_{\text{lw}} = 6.6$ mV.

6. Estimation of the lightwave-induced bias under cw illumination

To estimate the peak lightwave induced bias in the STM junction we first approximate the peak far-field strength of the MIR radiation in the focus of our parabolic mirror inside the STM (focus length: 18.64 mm). The beam propagated into the microscope has a power of 5 mW at a wavelength of 10 μm and a gaussian intensity distribution with a full-width at half-maximum radius of 4 mm. Considering the angle of incidence of the beam with respect to the surface normal of the sample (60°), we extract a peak far-field strength of 0.625 kV/cm.

To capture the coupling of the far field to the near field via the metallic tip, we refer to the numerical simulations using the frequency-domain finite element solver COMSOL published in [21]. The transfer function features an approximately 1/frequency dependence in amplitude. For the wavelength used in the experiment and a tip-sample separation of 1 nm we extract a field enhancement on the order of 10^2, resulting in a peak near-field strength of ~0.01 V/nm. We therefore estimate the peak lightwave-induced bias to be on the order of 10 mV. As our microscope is not configured for high sensitivity in scanning tunneling spectroscopy, this bias lies beyond our spectral resolution capabilities.

7. Background-free near-field detection at small oscillation amplitudes

The scattered intensity I is proportional to the squared magnitude of the scattered electric field $|E|^2$, where the field can be expanded as $E(t) = \sum_n E_n \exp(i2\pi n\nu t)$, with E_n denoting the complex Fourier coefficients, ν the tip oscillation frequency, and t the time [24]. Since the fundamental component E_0 is typically much larger than the higher-order terms, the dominant contribution to the second harmonic intensity component I_2 arises from the term $\propto E_0^* E_2 + E_0 E_2^*$ [24, 8].

In our experiments, the oscillation amplitude $A \approx 100$ pm as well as the lateral (3 nm) and vertical (500 pm) scan range are several orders of magnitude smaller than the mid-infrared wavelength (~10 μm). Under these conditions, E_0 can be assumed as spatially constant, such that I_2 predominantly reflects changes in E_2 rather than being influenced by far-field artifacts.

Experimentally, we support this conclusion by recording the distance-dependent near-field signal for an oscillation amplitude of A = 1 nm and over a tip retraction distance of 4.5 nm (Fig. S2) – values much larger than the ones used in the experiments shown in the main text. Even in this regime, we see that the pseudo-heterodyne signal s_2 and the bare intensity-resolved signal I_2, both demodulated at the second harmonic of the tip oscillation frequency, align with good agreement, confirming that variations in background interference do not contribute significantly to the scattered optical signal.

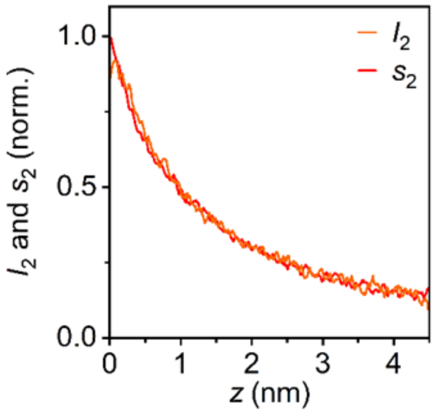

Figure S2. Background-free detection of near fields at small tip oscillation amplitudes. Comparison between the optical signal amplitude s_2 (red) extracted using pseudo-heterodyne detection and the intensity I_2 (orange), both demodulated at the second harmonic of the tip oscillation frequency, for a varying tip-sample distance z. For the oscillation amplitude of A = 1 nm used here, s_2 virtually follows I_2 over several nanometers of distance, indicating no pronounced influence of modulated background interference.

8. Statistical analysis of the modulation length of the NOTE signal

To assess the consistency of the modulation length reported in Fig. 3b, we extract eight additional step-edge profiles from different locations across the scan area, and fit each profile as described in the main text (Fig. S3). Our analysis shows a mean modulation width of 130 pm – matching the one extracted in Fig. 3b – with a standard deviation of 35 pm. This demonstrates consistent atomic-scale lateral confinement across multiple step edges.

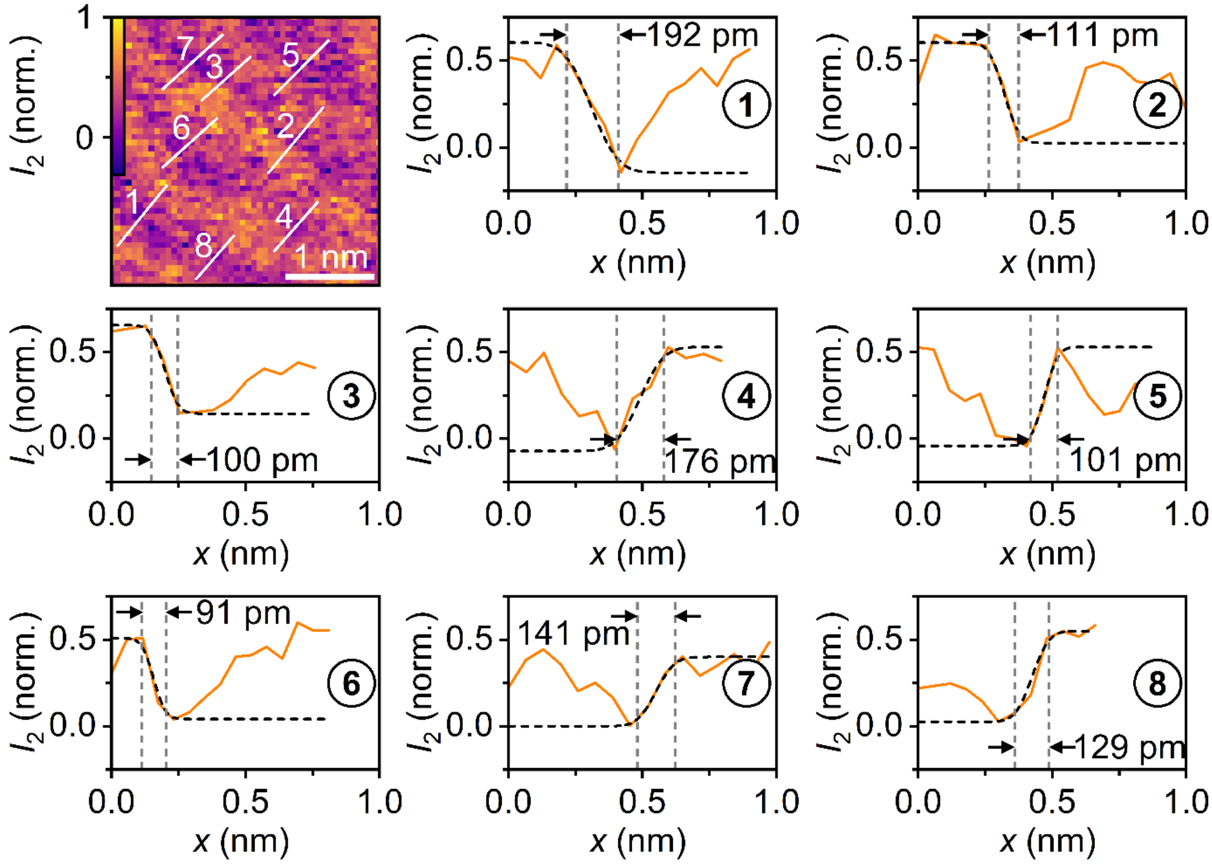

Figure S3. Statistics of the step-edge modulation width extraction. The plot shows multiple line profiles extracted from the 2D scan presented in Fig. 3b of the manuscript. Each line profile was independently fitted with an error function, and the lateral confinement was quantified by extracting the distance between the 90% and 10% intensity levels of the fitted step edge. This

statistical analysis yields a mean modulation width of 130 pm with a standard deviation of 35 pm, demonstrating consistent Ångstrom-scale spatial confinement.

9. Calibration of the tip oscillation amplitude

The tip is glued to a quartz sensor (qPlus [15]), whose deflection is measured by the induced piezoelectric voltage V_{osc} as measured with a high-frequency charge amplifier (Femto HQA-15M-10T). To determine the tip-oscillation amplitude A, this induced voltage signal is demodulated at the driving frequency of the sensor assembly $V_1 = V_{\mathrm{osc}}(\nu = \nu_{\mathrm{res}})$, which is continuously controlled to be at the instantaneous resonance frequency of the tuning fork ν_{res} by a phase locked loop. A PID control loop then adjusts the amplitude of the sinusoidal driving voltage V_{exc} for V_1 to be constant.

To calibrate the tip-oscillation amplitude A, we use an arbitrary value for the setpoint of V_1 and approach the tip in STM feedback mode (e.g. $I_{\mathrm{set}} = 100$ pA, $V_{\mathrm{set}} = 1$ V), recording the absolute position z_α of the vertical sample fine scanner piezo. We repeat the experiment with a different setpoint of V_1, approach to the same STM setpoint and again record the absolute position z_β of the vertical sample fine scanner piezo. Estimating the time-averaged tunneling current to be dominated by the point of closest approach during the oscillation cycle, a valid assumption for amplitudes $A \gg \kappa^{-1}$, the calibration factor δ is retrieved as $\delta = \frac{z_\beta - z_\alpha}{V_{1,\beta} - V_{1,\alpha}}$, where $V_{1,\alpha}$ and $V_{1,\beta}$ denote the setpoints of V_1 corresponding to z_α and z_β, respectively. Conceptually, this procedure transfers the calibration of the sample fine scanner piezo - established by imaging well-characterized surface features reported in literature - to the oscillation amplitude of the tip. Applying the same calibration factor to the piezoelectric voltage demodulated at the second harmonic of ν_{res}, $V_2 = V_{\mathrm{osc}}(\nu = 2\nu_{\mathrm{res}})$, yields the values for the second harmonic of the tip-oscillation frequency A_2 as discussed in the main text.

10. Excluding anharmonicity artefacts in the 2D scan of Figure 3

To investigate the relationship between I_2 and A_2 in the data presented in Figure 3 of the main text, we perform a pixel-by-pixel correlation analysis by plotting I_2 (spatial distribution in Fig. S4a) against the corresponding values of A_2 (spatial distribution in Fig. S4b) in a two-dimensional histogram (Fig. S4c). The resulting distribution shows no discernible correlation between the two signals - there is no systematic trend of increasing or decreasing I_2 with higher A_2 values. For a more quantitative assessment, we compute the Pearson correlation coefficient r [35]. In this dataset, we obtain $r = 0.091$. Since values of $r < 0.1$ are commonly interpreted as indicative of no significant correlation [35], we conclude that variations in A_2 do not have a measurable effect on I_2 under the conditions of this experiment.

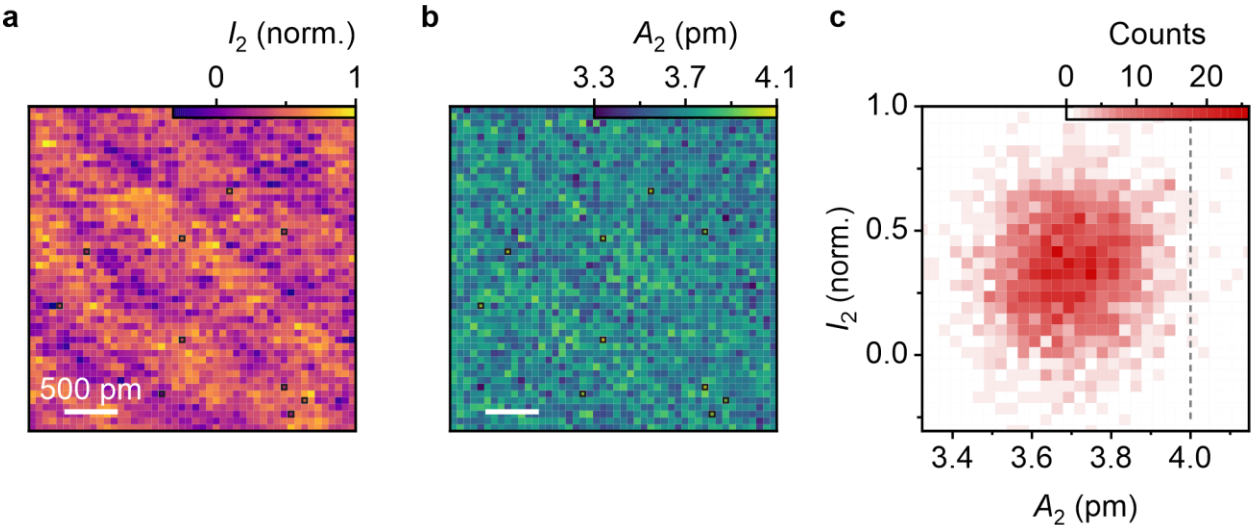

Figure S4. Negligible anharmonicity in the 2D scan of Figure 3. (a) Second-harmonic scattered intensity I_2 map of a vicinal gold surface, recorded in AFM feedback with an oscillation amplitude of A = 100 pm and a frequency shift setpoint of $\Delta\nu_{set}$ = -1.7 Hz. (b) Simultaneously acquired map of the local second-harmonic oscillation amplitude component A_2. In contrast to the behavior observed in Figure 4 of the main text, A_2 remains predominantly below the noise floor throughout

the scan, indicating minimal mechanical anharmonicity. (c) Two-dimensional histogram showing the pixelwise correlation between I_2 and A_2 from the maps in panels a and b. The color scale represents the number of pixels whose value pairs fall within each histogram bin. The highlighted areas in a and b show the 10 data points to the right of the grey dashed line in c, where $A_2 > 4$ pm.

11. Excluding anharmonicity artefacts as the origin of the optical signals in Figure 1

To exclude a significant contribution of the artefact mechanism associated with the second-harmonic mechanical oscillation A_2 to the optical signals in Figure 1d, we perform additional measurements under corresponding conditions. We once more investigate the distance dependence of the relevant signals at an oscillation amplitude of 250 pm and with minimized d.c. bias (Fig. S5). The tunneling current J exhibits the expected exponential decay for increasing tip-sample separation (Fig. S5a), while A_2 remains flat across the entire scan range (Fig. S5b), ensuring that the tip motion remains harmonic during the measurement. By fitting the optical sidebands SB_1 and SB_2 (Fig. S5c) with Bézier splines, we extracted the pseudo-heterodyne amplitude s_2 and phase ϕ_2 (Fig. S5d) [24]. The experiment shows the characteristic phase shift as well as the rapid rise in s_2, reproducing the behavior that was observed in Figure 1d, while the mechanical tip motion stays perfectly harmonic (i.e. A_2 stays unchanged), proving that these features do not stem from artefacts introduced by anharmonicity of the tip oscillation.

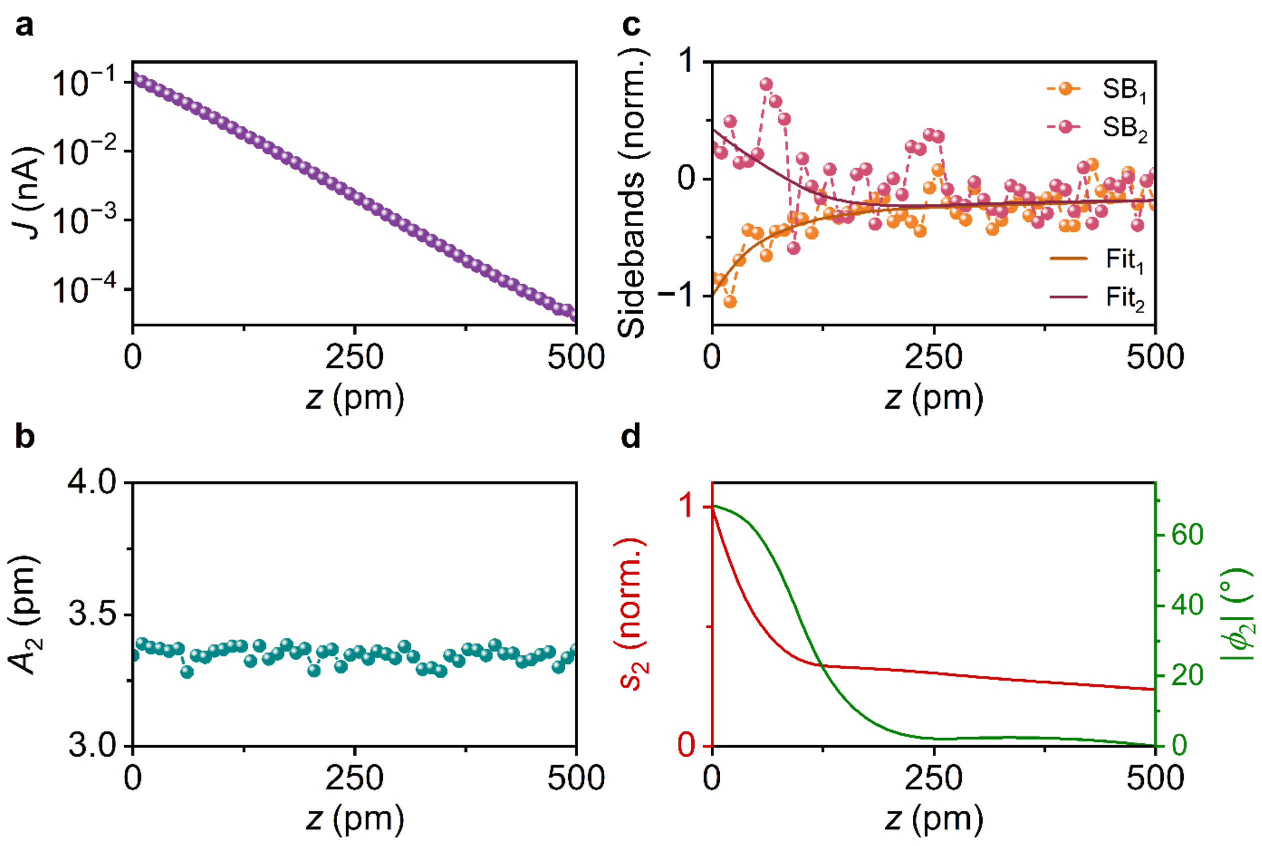

Figure S5. Retraction curves showing an optical phase shift while excluding the influence of anharmonicity artefacts. (a) Tunneling current J as a function of the relative tip-sample distance z (oscillation amplitude $A = 250$ pm, d.c. bias voltage V_{dc} minimized). (b) Mechanical tip oscillation amplitude A_2, demodulated at the second harmonic of the oscillation frequency. No increase at small tip-sample distances is observed. (c) Sidebands SB_1 and SB_2 of the pseudo-heterodyne detection scheme. Owing to the relatively low signal-to-noise ratio of the measurement, the experimental data (spheres) were fitted using Bézier spline interpolation (solid lines) for further analysis. d) Near-field amplitude s_2 (red) and magnitude of the relative phase $|\phi_2|$ (green), demodulated at the second harmonic of the tip oscillation frequency. The near-field amplitude s_2 is constructed from the fit as $\sqrt{\mathrm{SB_1}^2 + \mathrm{SB_2}^2}$ while the phase ϕ_2 is extracted by evaluating $\tan^{-1}\left(\frac{\mathrm{SB_1}}{\mathrm{SB_2}}\right)$ [24].

References.

[36] B. J. van Wees, H. van Houten, C. W. J. Beenakker, J. G. Williamson, L. P. Kouwenhoven, D. van der Marel, and C. T. Foxon, *Quantized conductance of point contacts in a two-dimensional electron gas*, Physical Review Letters **60**, 848-850 (1988).
